# Silicon Priming Regulates Morpho-Physiological Growth and Oxidative Metabolism in Maize under Drought Stress

**DOI:** 10.3390/plants8100431

**Published:** 2019-10-20

**Authors:** Abida Parveen, Wei Liu, Saddam Hussain, Jaleel Asghar, Shagufta Perveen, Yousheng Xiong

**Affiliations:** 1 Department of Botany, Government College University, Faisalabad 38000, Pakistan; abidauaf@yahoo.com (A.P.); jaleelasghar005@gmail.com (J.A.);; 2 Institute of Plant Protection and Soil Fertilizer, Hubei Academy of Agricultural Science, Wuhan 430064, China; weiliu8844@126.com; 3 Department of Agronomy, University of Agriculture, Faisalabad 38040, Punjab, Pakistan

**Keywords:** antioxidant machinery, chlorophyll pigments, drought stress, maize growth, osmoprotectants, silicon

## Abstract

Seed priming with silicon (Si) is an efficient and easy method to regulate plant tolerance against different abiotic stresses. A pot experiment was conducted to examine the Si-mediated changes in oxidative defense and some vital physio-biochemical parameters of maize under a limited water supply. For this purpose, two maize varieties (Pearl and Malka) with different Si priming treatments (0, 4 mM, 6 mM) were grown under a control and 60% field capacity for three weeks. At 60% field capacity, significant reductions in plant growth attributes and chlorophyll contents were recorded compared with the control. The negative effects of drought stress were more severe for Malka compared with Pearl. Drought stress increased the malondialdehyde (MDA) and hydrogen peroxide (H_2_O_2_) contents, altered the activities of antioxidant enzymes (superoxide dismutase (SOD), peroxidase (POD), and catalase (CAT)), and triggered the accumulation of soluble sugars, glycine betaine, proline, and phenolics contents. Nevertheless, seed priming with silicon at 4 or 6 mM was effective in alleviating the detrimental effects of drought stress in both cultivars. Si priming particularly at 6 mM significantly enhanced the shoot and root lengths as well as their biomass and improved the levels of photosynthetic pigments. Moreover, Si treatments enhanced the activities of antioxidant enzymes (SOD, POD, and CAT) while it reduced the MDA and H_2_O_2_ contents in both cultivars under stress conditions. In crux, the present investigation suggests that Si priming mitigates the harmful effects of drought stress and contributes to the recovery of maize growth.

## 1. Introduction

Drought stress is considered as the most important abiotic stress that hampers the growth and development of plants resulting in severe yield losses [1]. Drought is known to disrupt the integrity of membrane, chlorophyll contents, water relations, osmotic adjustment, and photosynthetic activity in a number of crops [2,3,4]. However, adaptation of plants to water deficit conditions occurs as a result of different events and processes mainly including changes in growth pattern, plant structure, physio-biochemical processes, osmotic potential, and antioxidant defense system [2]. In maize, drought stress led to the impairment of growth traits including plant height, leaf area, number of leaves per plant, and shoot biomass [5]. Drought-induced reductions in fresh and dry biomass production in some crop plants have been well reported in the past [2,3]. Drought also causes changes in the oxidative defense system, synthesis of photosynthetic pigments, and accumulation of lipids and proteins [2]. Akram et al. [6] documented that drought stress reduced the accumulation of chlorophyll pigments in canola, which ultimately affected the use of energy and harvesting of light by the plants. The loss of chlorophyll is the leading cause of inactivation of the photosynthetic process due to water loss. Drought stress has been reported to decrease chlorophyll pigments in many crops such as carrot [7], chickpea [8], and potato [9]. 

Drought stress causes the overproduction of reactive oxygen species (ROS) and damages the membrane in two maize hybrids [10]. In tomato, the accumulation of hydrogen peroxide (H_2_O_2_) and lipid peroxidation rate (malondialdehyde (MDA) contents) were increased under drought stress [11]. In order to overcome the excessive accumulation of ROS and protect from oxidative damage under drought stress, plants possess a very sophisticated and efficient defense system comprising of different enzymatic (e.g., peroxidase (POD), superoxide dismutase (SOD), and catalase (CAT)) and non-enzymatic (e.g., phenolics) antioxidants [12,13]. Yadav and Sharma [14] reported that significant improvement in the activities/levels of enzymatic and non-enzymatic antioxidants occur in plants to overcome the drought stress. In radish, the activities of oxidative enzymes increased under drought stress [15]. Moreover, some higher plants begin to accumulate osmolytes such as proline, glycine betaine, soluble sugars, and many secondary metabolites to combat the drought stress condition [1,2,5].

Silicon (Si) is one of the major elements used to alleviate the adverse effects of drought in plants. The Si application has been reported to improve plant tolerance against drought in a number of field crops including rice [16], sorghum [17], maize [18], wheat [19], sunflower [20], cucumber [21], soybean [22], and tomato [23]. Seed priming with Si is an efficient and easy method to regulate plants’ tolerance against drought stress and it is widely used in many plants [24,25]. The Si priming confers drought tolerance in plants by improving the activities of various enzymes and enhancing physiological growth and biomass production [16,19,20]. Under drought stress, Si is beneficial to prevent transpirational loss, and improve many physiological and biochemical processes in plants. Hamayun et al. [26] reported that Si regulates many processes which improve the water status of plants, enhance photosynthetic activity, and strengthen the structure of leaf organelles. The application of Si reduced lipid peroxidation and H_2_O_2_ accumulation in seedlings of rice under drought stress [16]. The protective effects of Si against oxidative damage have been documented in different crop plants [16,17,18,27].

Maize is considered as one of the most important crops of the world [28]. It is a local crop in China, India, Thailand, and Pakistan. The protective effects of the exogenous Si applied as foliar application or in rooting medium are present in the literature [29], however, the role of Si as pre-sowing seed treatment in maize is not well established and needs further studies. Therefore, the present experiment investigated the influence of the exogenous application of Si as seed priming on maize growth under drought stress. The specific objectives were (a) to ascertain the Si priming-mediated changes in morphological attributes, oxidative defense system, photosynthetic pigments, and osmolyte accumulation in order to enhance drought tolerance in maize; and (b) to examine the morpho-physiological and biochemical response of two maize cultivars to drought stress and Si priming. 

## 2. Materials and Methods

### 2.1. Plant Material and Treatments

The experiment was conducted during March 2019 at the Department of Botany, Government College University Faisalabad, Pakistan under natural climatic conditions (average day and night temperatures were 39.2 ± 4 °C and 23.5 ± 5 °C, respectively). The relative humidity ranged from 31.6% to 65.8%, and day length from 11–12 h. Seeds of two cultivars cv. Pearl and cv. Malka were obtained from Ayub Agricultural Research Institute (AARI) Faisalabad, Pakistan and were primed in a solution of 0, 4, and 6 mM sodium metasilicate (Na_2_SiO_3_; pH 11.76; 200 g seeds of each variety were soaked in 250 mL solution of 0, 4, and 6 mM levels, respectively) for 16 h. Ten maize seeds were sown in plastic pots (20 cm internal diameter and 25 cm height) containing 10 kg loamy soil having electrical conductivity (EC) 1.31 dS m^−1^, TSS (total suspended solids) 13.10 me L^−1^, CO_3_ 0.84 me L^−1^, HCO_3_ 4.84 me L^−1^, Cl^-^ 5.84 me L^−1^, Na^+^ 6.24 me L^−1^, Ca^2+^ and Mg^2+^ 5.88 me L^−1^ and sodium adsorption ratio (SAR) 3.84 mmol ^1/2^ L^1/2^. After complete germination, four equal sized seedlings were kept per pot. Ten-day-old seedling were subjected to two different moisture regimes (60% field capacity as drought stress, and 100% field capacity as a control). Soil moisture was calculated on soil dry weight basis. The pots were weighed in two-day intervals to compensate the water loss by evapotranspiration; therefore, the pot soil moisture was kept at 100% and 60% field capacity according to treatments. The saturation of the soil used was 32% and pH 8.65. The plants were allowed to grow for 10 days before the start of water deficit conditions. The soil moisture content level was controlled by adding water daily according to field capacity. Data for various growth and biochemical parameters was recorded after three weeks of stress imposition. 

### 2.2. Parameters

Growth attributes: Two plants per pot were harvested and data for shoot and root lengths and fresh and dry weights were recorded. For dry weight, plants were put in an oven at 68 °C for one week, and plants were dried until constant weight.

Photosynthetic pigments: Data regarding chlorophyll and carotenoid contents in maize were recorded according to the method of Lichtenthaler and Wellburn [30]. Briefly, 0.05 g fresh leaf sample of maize was placed in 10 mL 80% acetone (v/v), and the optical density of the extract was recorded at 663, 645 and, 480 nm for chlorophyll a, chlorophyll b, and carotenoids, respectively.

Osmolyte accumulation: Leaf free proline content in maize was recorded using the method of Bates et al. [31]. Fresh leaf sample was standardized by using sulfosalicylic acid (3% w/v), and then the mixture was filtered with the help of filter paper. After that ninhydrin and glacial acidic acid were added to this mixture and heated at 100 °C for 1 h in a water bath. Later, toluene was added, and absorbance was calculated at 520 nm. 

The glycine betaine (GB) contents in fresh leaf samples were determined by using the protocol of Grieve and Grattan [32]. In total, 500 mg leaf sample was crushed with 0.5% of toluene (10 mL) and then filtered into the mixture. The absorbance of samples was read at 395 nm spectrophotometrically. 

For recording total soluble sugar, fresh maize leaf samples were frozen at −10 °C and were later ground in 0.1 M monobasic phosphate buffer. The extracts were filtered and centrifuged in cold centrifuge at 3000 rpm for 15 min. Phenol-sulfuric acid method was used to find out total soluble sugars present in samples using the method of Dubois et al. [33].

Malondialdehyde (MDA): A 0.5 g frozen leaf sample of maize was squeezed into fine powder. Then, 5 mL of 10% trichloroacetic acid (TCA) solution for each sample were used during grinding. Then, this material was centrifuged at 7000 rpm for 15 min. After centrifugation, the supernatant was used to analyze the MDA by using the protocol of Zhang [34]. 

Hydrogen peroxide (H_2_O_2_): The H_2_O_2_ contents were examined by using the TCA (0.1% w/v) method by following the procedure of Alexieva et al. [35]. Briefly, 500 mg leaf tissue was placed in an ice bath with 5 cm^3^ TCA. This mixture was centrifuged at 12,000 rpm for 15 min and then 0.5 mL of supernatant was mixed with 0.5 mL of potassium phosphate buffer (100 mM) and 1 mL potassium iodide (1M). After that absorbance was checked at 390 nm.

Enzymatic and non-enzymatic antioxidants: A 500 mg leaf sample from each replicate was extracted in 10 mL of potassium phosphate buffer. The POD and CAT enzyme activities were analyzed by using the procedure of Chance and Maehly [36]. The reaction mixture for CAT (3 mL) contained 50 mM phosphate buffer (pH 7.8), 5.9 mM H_2_O_2_, and 0.1 mL enzyme extract. Absorbance change at 240 nm was measured after every 20 s until one min. One unit of CAT was considered as 0.01 absorbance change per min. The POD reaction mixture consisted of 50 mM potassium phosphate buffer (pH 7.8), H_2_O_2_ (40 mM), guaiacol (20 mM), and enzyme extract 0.1 mL. The absorbance change was measured after every 20 s interval at 470 nm for one min. One unit of POD was an absorbance change of 0.01 in one min. The SOD activity was estimated according to Giannopolitis and Riess [37]. Then, 50 µM NBT (nitro-blue tetrazolium chloride, Sigma-Aldrich, St. Louis, MO, USA), 1.3 µM riboflavin (Sigma-Aldrich, St. Louis, MO, USA), 13 mM methionine (Sigma-Aldrich, St. Louis, MO, USA, 75 µM EDTA (Ethylenediaminetetraacetic acid, Sigma-Aldrich, St. Louis, MO, USA, and 50 mM phosphate buffer were added in 20–50 µL of sample for SOD. Test tubes filled with this solution and irradiated under light at 78 µmol m^−2^s^−1^ for 15 min and reading was taken at 560 nm.

Total phenolics of the fresh leaf sample were determined by using the protocol of Julkunen-Titto [38]. A gallic acid calibration curve that ranged from 10–80 mg L^−1^ was used to calculate total phenolics of the maize leaf sample. 

### 2.3. Statistical Analysis

The experiment was arranged in a completely randomized design under factorial arrangement and all the treatments were replicated three times. Experimental data on all variables were subjected to analysis of variance (ANOVA) using COSTAT software. The difference among means was calculated with a least significant difference at 5% probability level. 

## 3. Results

### 3.1. Growth Attributes

**Figure 1:**
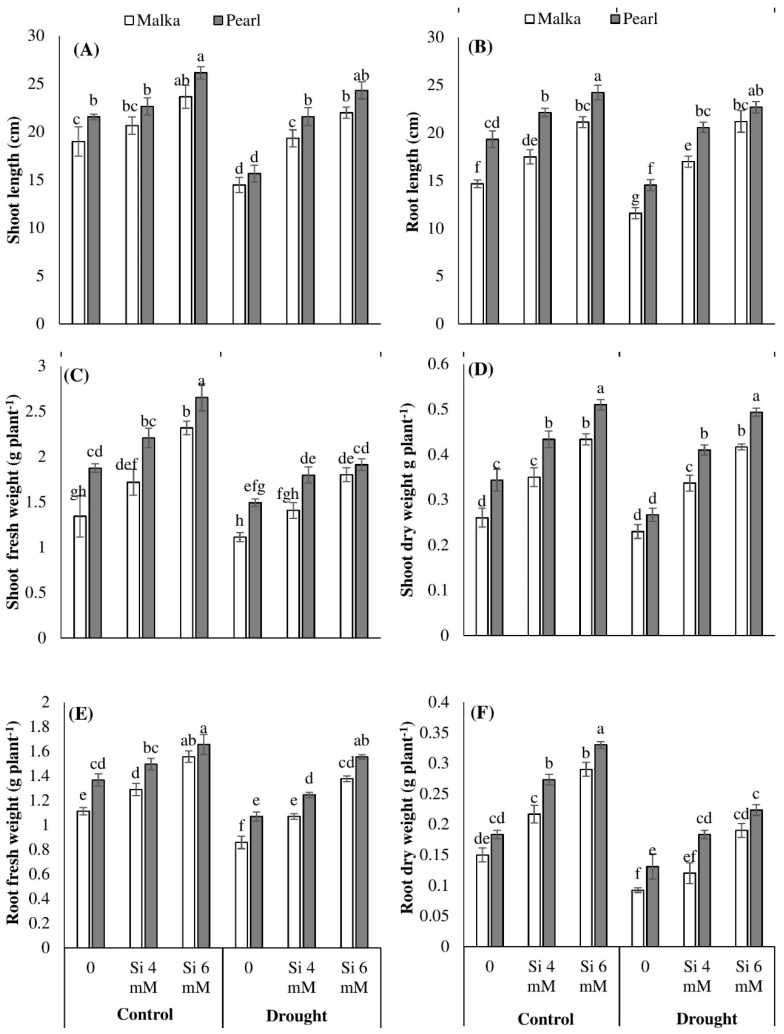
Effect of seed priming with silicon on (**A**) shoot length, (**B**) root length, (**C**) shoot fresh weight, (**D**) shoot dry weight, (**E**) root fresh weight, and (**F**) root dry weight of two maize cultivars under drought stress. Mean with same letter(s) do not differ significantly at *p* < 0.05. Error bars above the means indicate standard error (*n* = 3).

Drought stress (60% field capacity) was found to significantly (*p* ≤ 0.001) reduce the shoot and root length as well as their fresh and dry weights in both maize cultivars, compared with the control. Exogenous application of both Si levels (4 and 6 mM) significantly (*p* ≤ 0.01) mitigated the detrimental effects of drought stress in all growth attributes of both maize cultivars. Nevertheless, such positive effects were more prominent for 6 mM Si. Averaged across two cultivars, 6 mM Si increased the shoot length of maize by 22.95% and 53.70%, root length by 34.72% and 69.42%, shoot dry weight by 57.61% and 83.07%, and root dry weight by 86.66% and 88.35% under control and drought stress conditions, respectively, compared with no Si application. The cv. Pearl performed better than cv. Malka regarding all growth parameters (Figure 1; Table 1).

### 3.2. Photosynthetic Pigments 

**Figure 2:**
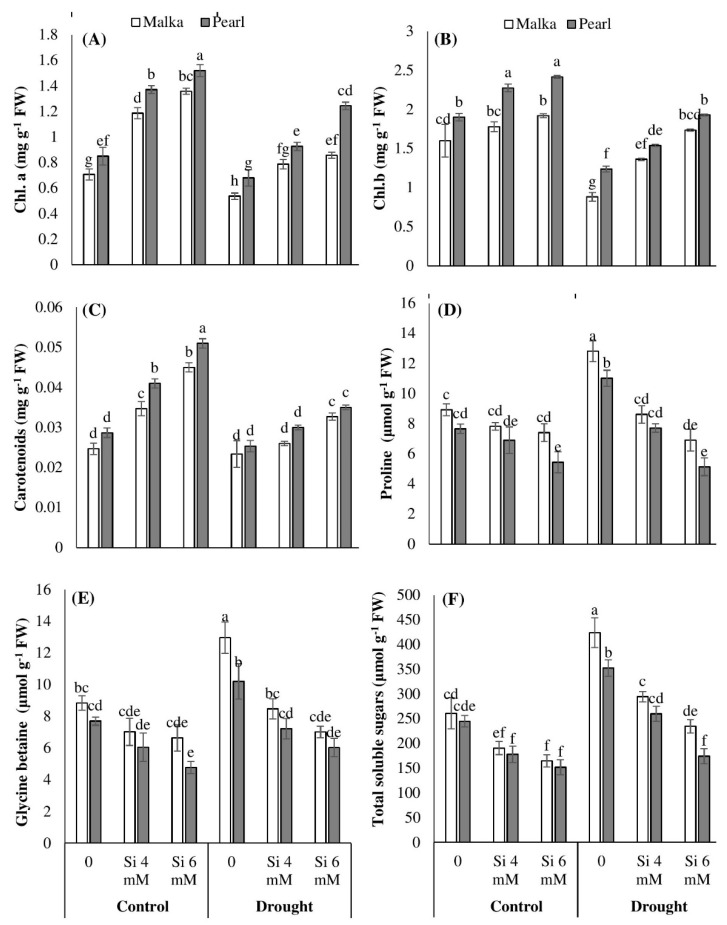
Effect of seed priming with silicon on (**A**) chlorophyll a, (**B**) chlorphyll b, (**C**) carotenoids, (**D**) proline, (**E**) glycine betaine, and (**F**) total soluble sugar contents in two maize cultivars under drought stress. Mean with same letter(s) do not differ significantly at *p* < 0.05. Error bars above the means indicate standard error (*n* = 3).

Results show that chlorophyll (Chl) a, b, and carotenoids were significantly (*p* ≤ 0.001) reduced under drought stress in both maize cultivars. Moreover, the difference between cultivars regarding accumulation of chlorophyll contents was apparent, the cv. Pearl recorded higher chlorophyll a, b, and carotenoid contents as compared with cv. Malka. Seed priming with Si significantly (*p* ≤ 0.001) increased chlorophyll contents in both cultivars under control and drought conditions. Averaged across two cultivars, Si priming at 6 mM increased the Chl a content by 85% and 71%, and Chl b content by 24% and 76% under control and drought stress conditions, respectively, compared with no Si application. The cv. Pearl recorded a greater increase in chlorophyll pigments in both stressed and non-stressed conditions. The interaction between drought stress and silicon application was significant (*p* ≤ 0.001) for Chl b and carotenoid contents (Figure 2A,B; Table 1).

### 3.3. Osmolyte Accumulation

Data revealed that proline contents were significantly (*p* ≤ 0.001) increased in maize plants under drought stress conditions (Table 1). Pre-sowing seed treatment with Si significantly (*p* ≤ 0.001) decreased proline levels in both cultivars in stressed and non-stressed conditions. However, 6 mM pre-treated seeds showed a significantly higher decrease in proline accumulation compared with 4 mM Si. Between the two cultivars, the cv. Malka accumulated more proline as compared with cv. Pearl (Figure 2D).

Glycine betaine and total soluble sugars were significantly (*p* ≤ 0.001) increased in both cultivars under drought stress. However, Si priming was found to significantly (*p* ≤ 0.001) decrease the accumulation of glycine betaine and total soluble sugars in stressed and non-stressed plants (Figure 2E,F; Table 1). Silicon at 6 mM recorded a greater decrease in these attributes than 4 mM. Cultivar difference was also evident, cv. Malka showed higher accumulation of glycine betaine and total soluble sugars in all osmoprotectants (Figure 2; Table 1).

### 3.4. Oxidative Stress Attributes

**Figure 3:**
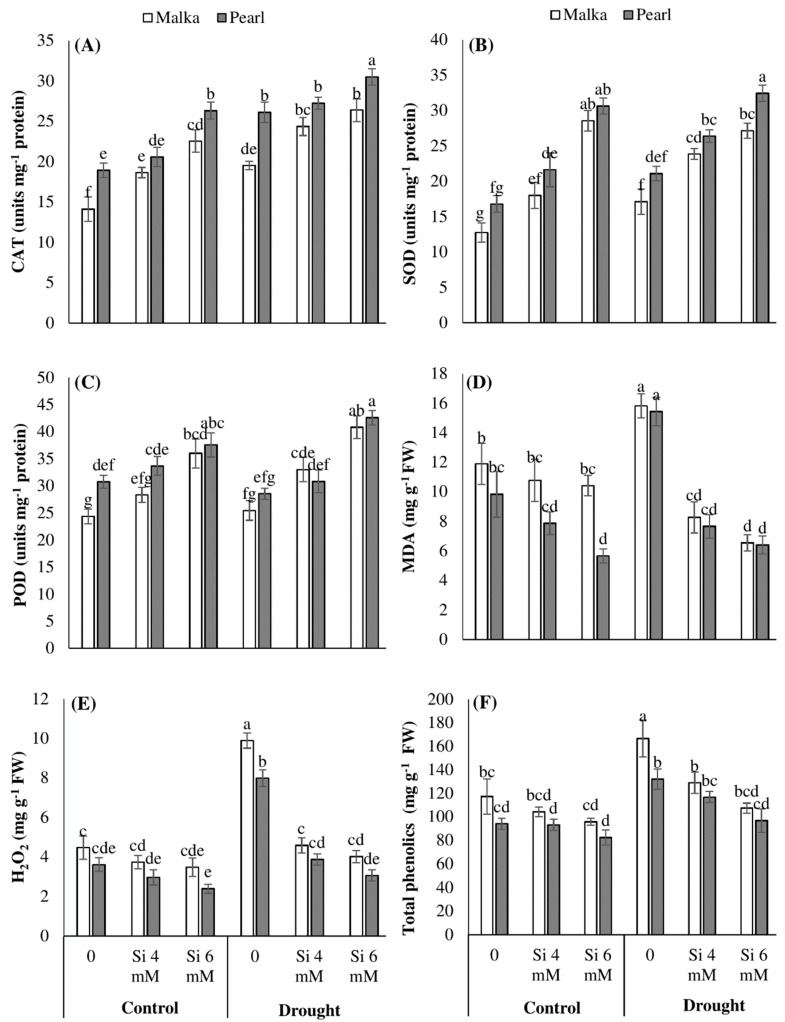
Effects of seed priming with silicon on (**A**) catalase, (**B**) superoxide dismutase, (**C**) peroxidase, (**D**) malondialdehyde, (**E**) hydrogen peroxide, and (**F**) total phenolics content in two maize cultivars under drought stress. Mean with same letter(s) do not differ significantly at *p* < 0.05. Error bars above the means indicate standard error (*n* = 3).

The activities of CAT and SOD were significantly (*p* ≤ 0.001) increased under drought stress conditions in both cultivars (Figure 3A,B; Table 1). The Si seed priming (4 and 6 mM) further increased the SOD and CAT activities to a significant (*p* ≤ 0.001) level in both cultivars under control and drought stress conditions. The Si at 6 mM greatly enhanced the activities of both these antioxidants in maize cultivars with the range of 16%–124% under control and drought stress, compared with no Si treatment. A non-significant effect of drought stress was recorded regarding POD activity, while Si pre-treated seeds significantly enhanced the POD activity in both cultivars under stress and control conditions (Figure 3C; Table 1). Compared with no Si application, Si priming at 6 mM increased the POD activity of both maize cultivars by 35% and 55%, under control and drought stress conditions, respectively. Cultivar difference was also evident as Pearl recorded higher activities of antioxidants than Malka which was sensitive against drought stress (Figure 3; Table 1).

A non-significant increase was found in the MDA content of maize cultivar when subjected to drought stress conditions. Exogenous application of Si significantly (*p* ≤ 0.001) inhibited MDA accumulation in stressed and non-stressed conditions (Figure 3D; Table 1). An increase (*p* ≤0.001) in H_2_O_2_ contents in both cultivars was recorded under drought stress. However, the response of cultivars was significantly (*p* ≤ 0.01) different, and Malka appeared more sensitive to cultivars than Pearl (Figure 3D, Table 1). The interaction between stress and Si treatments was significant (*p* ≤ 0.001). Silicon seed priming at 4 or 6 mM was found to significantly decrease the accumulation of MDA and H_2_O_2_ in both maize cultivars under drought stress. Out of both Si levels, 6 mM performed better than 4 mM silicon (Figure 3; Table 1).

When maize plants were subjected to drought stress, a significant (*p* ≤ 0.001) increase in phenolics accumulation was observed in both cultivars under stressed and non-stressed conditions. Seed-priming with silicon (mainly 6 mM) significantly (*p* ≤ 0.001) decreased phenolic contents in both cultivars under drought stress and control conditions. Cultivar difference was also evident as cv. Pearl showed higher phenolic contents as compared with cv. Malka (Figure 3E; Table 1).

## 4. Discussion

The present experiment investigated the influence of the Si priming on morphological attributes, oxidative defense system, photosynthetic pigments, and osmolyte accumulation in two maize cultivars under drought stress. Drought stress at 60% field capacity was found to significantly reduce the root and shoot growth attributes of non-primed seedlings in the range of 11%–38% for both cultivars (Figure 1). Drought-induced reductions in seedling growth attributes might be due to disturbances in stomatal functioning and root architecture which hinder the supply of water and nutrients for normal metabolic processes in plants [3,4,5,10,39]. In the past, several researchers have documented that drought-induced stomatal limitations altered the metabolic pathways in plants through reduced CO_2_ and nutrient uptake [2,3,40,41]. Between the two cultivars, Pearl recorded better shoot and root growth than Malka under control as well as drought stress conditions. In non-primed seedlings, drought stress decreased the root:shoot ratio by 30% in Malka and 8% in Pearl, which was the result of poor growth performance and lesser tolerance in the Malka cultivar to drought stress (Figure 1). 

Seed priming with Si enhanced the seedling growth attributes of both maize cultivars under the control as well as drought stress conditions (Figure 1). However, Si-induced increases in maize growth attributes were more prominent under drought stress compared with the control. Across two maize cultivars, 6 mM Si priming averagely increased the seedling growth attributes of maize by 48% and 65% under the control and drought stress conditions, respectively (Figure 1). Better growth of maize seedlings developed from primed seeds was possibly due to early and fast completion of pre-germination metabolic events during the priming process [1,12,42]. During germination, seed priming-induced regulation of hydrolases and other metabolic enzymes increases the synthesis of metabolites [12,42], which result in earlier and uniform seedling growth. Averaged across different stress treatments, Si priming at 6 mM increased the root:shoot ratio by 14% and 6% in Malka and Pearl cultivars, respectively (Figure 1). These results relate to the study of Liu et al. [43] and Kaya et al. [44] in which biomass was increased in alfalfa and maize seedlings treated with Si under drought stress, respectively. 

In the present study, drought stress decreased Chl. a and Chl. b contents, while carotenoid contents remain unchanged in both maize cultivars (Figure 2). The decrease in chlorophyll content is an indicator of oxidative stress and the response of pigment photo-oxidation as well as degradation of chlorophyll contents [4,5,45]. The drought-induced reduction in chlorophyll contents of non-primed maize seedlings might be due to overproduction of ROS and higher oxidative damage (Figure 3). Manivannan et al. [45] and Hussain et al. [3] documented that drought stress decreased the photosynthetic pigments in sunflowers due to excessive production of ROS, inefficient nutrient uptake by the plants, and disturbance in activities of enzymes at cellular levels. Our results revealed that Si priming enhanced the chlorophyll contents in both maize cultivars. Compared with no Si application, 6 mM Si priming increased chlorophyll contents by 55% and 74% under control and drought stress (Figure 2). These results might be attributed to Si-induced enhancement in seedling vigor, an increase in the antioxidant defense system, and alleviation of oxidative damage in primed seedlings (Figure 1, Figure 2 and Figure 3). Previously, Dehghanipoodeh et al. [46] also reported Si-mediated enhancement in chlorophyll and growth parameters in strawberry cultivars under control and water-deficit conditions. Various other researchers also documented that Si mediated an increase in the activities of various antioxidant enzymes (such as SOD and CAT), and improved chloroplast ultrastructure and membrane stability which help maintain the contents of photosynthetic pigments under stressful environments [47,48,49]. 

In the present study, proline, soluble sugars, and glycine betaine concentrations increased under drought stress (Figure 2). The increased concentration of these osmolytes in plants might be involved in different processes such as detoxification of ROS, membrane integrity, and stabilization in activities of various enzymes and osmotic adjustment in order to induce drought tolerance in plants [50]. Wu et al. [51] reported that accumulation of proline increased in drought-tolerant cotton plants that showed osmotic adjustment under drought stress. However, Pei et al. [19] considered an increased accumulation of osmolytes (e.g., proline) as a symptom of stress injury rather than an example of their role in tolerating stress. In the current study, Si priming was found to decrease the accumulation of osmolytes in maize seedlings particularly under stress conditions. Under drought stress, Si priming at 4 mM or 6 mM significantly decreased the proline accumulation in both maize cultivars compared with no Si application (Figure 2). The decrease in proline accumulation under drought stress after Si priming might be an indication of stress relief and alleviation of stress damage. Consistent with our results, several researchers have previously reported that Si application decreases the proline levels in wheat [19,52], sorghum [53], soybean [54], and rice [55] under stress conditions. 

In our study, drought stress was found to trigger the H_2_O_2_ contents in maize (Figure 3). The overproduction of ROS under drought stress caused a higher accumulation of MDA. Furthermore, the increase in H_2_O_2_ revealed that oxidants other than this might have been produced which caused the enhancement in H_2_O_2_ under drought stress [56]. Previously, several pieces of research have reported that drought increased the MDA and H_2_O_2_ concentrations in wheat [39], cucumber [21], and canola [6]. MDA is an indicator of oxidative damage in plants due to drought stress [13]. MDA works as a marker to point out lipid peroxidation under stress conditions and accumulation of ROS caused the instability of the membrane reflecting lipid peroxidation. Further, lipid peroxidation, membrane deteriorations, as well as degradation of nucleic acid and proteins take place due to free radicals that generate under drought stress causing the main reason of oxidative damage [2,56,57]. In this study, the activities of SOD and CAT were also triggered under drought stress (Figure 3). SOD functions as the first line of defense scavenging superoxide radicals (O_2_^•−^) to H_2_O_2_ which is further detoxified by POD, CAT, and ascorbate peroxidase (APX) [56]. An intricate antioxidant defense system with significant contributions of enzymatic (SOD, POD, CAT, and APX) and non-enzymatic antioxidants (e.g., phenolics) is involved in the detoxification of ROS. In the current study, drought stress significantly enhanced the accumulation of H_2_O_2_ in non-primed maize seedlings, while the activities of POD were not altered significantly for both cultivars. This clearly indicates that the H_2_O_2_-scavenging system through POD was poor in non-primed seedlings under drought; therefore, H_2_O_2_ accumulation in drought stress seedlings was 122% higher than the control (Figure 3). Our results are consistent with the findings of Wang et al. [49] who reported that POD activity remained unchanged in rice plants after 15 days of drought stress. Egert and Tevini [58] also reported that APX activity was enhanced in *Allium schoenoprasum* under drought stress, while POD activity remained unchanged revealing the minimal contribution of POD in H_2_O_2_ detoxification.

Maize seedlings emerged after Si priming had significantly lesser ROS concentrations and lipid peroxidation rate in comparison to non-primed seedlings (Figure 3) which indicated that oxidative stress and seedling damage induced by drought stress were efficiently overcome by Si priming. Compared with the control, the levels of H_2_O_2_ and MDA in Si-primed seedlings were statistically alike or even lesser under drought stress conditions. The Si priming triggered the activities of CAT, SOD, and POD under control and drought stress conditions, which were concomitant with the lower accumulations of H_2_O_2_ and MDA in maize under these treatments (Figure 3). Sayad and Gadallah [59] reported that Si application strengthened the antioxidative defense system and decreased lipid peroxidation and oxidative damage in maize under drought stress. Feng et al. [59] also concluded that application of Si enhanced the activities of antioxidant enzymes in cucumber. In the present study, the total phenolics contents in Si-primed seedlings were comparatively lower than non-primed seedlings, which might be attributed to lower ROS production in Si priming treatments (Figure 3). Reddy et al. [60] reported that an increase in total phenolic content correlates with the production of ROS in plants, and the duration and severity of stress. In crux, enhancement in the activities of antioxidant enzymes after Si priming kept a balance between ROS production and ROS detoxification, thereby protecting the oxidative damage (Figure 3). All the antioxidants triggered by seed priming acted coordinately in eliminating the ROS, which are vital for enhancing plant tolerance against drought stress [2,3,10].

## 5. Conclusions

Seed priming with Si was effective in mitigating the detrimental effects of drought stress in maize. Si priming, particularly at 6 mM, significantly enhanced growth attributes and improved the levels of photosynthetic pigments particularly in Pearl. Moreover, Si treatments reduced the MDA and H_2_O_2_ contents while it enhanced the activities of antioxidant enzymes (SOD, POD, and CAT) in both cultivars, which helped the crop to cope with drought stress. 

## Figures and Tables

**Table 1:** Summary of ANOVA regarding the effect of silicon priming on growth, photosynthetic pigments, and oxidative defense in two maize cultivars under drought stress conditions.

**Source of Variance**	**df**	**Shoot Length**	**Root Length**	**Shoot Fresh Weight**	**Shoot Dry Weight**	**Root Fresh Weight**	**Root Dry Weight**
Varieties (V)	1	41.38***	104.04***	1.24***	0.07***	0.31***	0.21***
Drought stress (S)	1	66.69***	32.49***	1.68***	0.01***	0.42***	0.32***
Treatments (T)	2	121.77**	160.70***	1.54***	0.11***	0.57***	0.27***
V × S	1	0.40 ns	4.84 ns	0.06 ns	8.03 ns	1.11 ns	0.06 ns
V × T	2	0.21 ns	2.79 ns	0.05 ns	3.08 ns	0.01 ns	0.06 ns
S × T	2	14.22**	9.37**	0.09 ns	0.01 ns	0.02 ns	0.09 ns
V × S × T	2	0.53 ns	0.09 ns	0.01 ns	4.52 ns	0.01 ns	0.01 ns
Error	24	-	-	-	-	-	-
**Source of Variance**	**df**	**Chl. a**	**Chl. b**	**Carotenoids**	**Free Proline**	**Glycine Betaine**	**Soluble Sugars**
Varieties (V)	1	0.34***	1.02***	1.52***	18.66***	20.14**	10,748.97 **
Drought stress (S)	1	0.96***	2.57***	6.93***	16.22***	29.51***	75,283.69 ***
Treatments (T)	2	0.95***	1.08***	7.13***	45.85***	46.30***	59,637.53 ***
V × S	1	0.01 ns	0.08*	1.61 ns	0.03 ns	0.26 ns	3909.85 ns
V × T	1	0.02 ns	2.53 ns	3.53 ns	0.71 ns	0.55 ns	310.50 ns
S × T	2	0.05***	0.09**	1.08***	12.80***	5.25*	5936.15 **
V × S × T	2	0.02 ns	0.03 ns	5.83 ns	0.11 ns	1.16 ns	232.79 ns
Error	24	-	-	-	-	-	-
**Source of Variance**	**df**	**Total Phenolics**	**MDA**	**H_2_O_2_**	**SOD**	**POD**	**CAT**
Varieties (V)	1	2747.44**	29.30**	9.82	116.31***	63.55*	145.53***
Drought stress (S)	1	6547.60***	3.46 ns	40.79***	97.12***	28.14 ns	272.29***
Treatments (T)	2	3060.95***	117.96***	36.42***	491.22***	444.07***	137.97***
V × S	1	23.89 ns	18.46*	0.18 ns	1.01 ns	28.27 ns	2.40 ns
V × T	2	291.20 ns	1.13 ns	0.31 ns	0.65 ns	9.77 ns	2.40 ns
S × T	2	723.26 ns	38.84***	17.47***	22.13 *	24.14 ns	4.95 ns
V × S × T	2	39.64 ns	1.77 ns	0.32 ns	3.86 ns	11.22 ns	0.39 ns
Error	24	-	-	-	-	-	-

*, **, *** = significant at *p* < 0.05, *p* < 0.01, and *p* < 0.001 levels, respectively. df = degrees of freedom; Ns = non-significant; Chl. a = chlorophyll a, Chl. b = chlorophyll b; H_2_O_2_ = hydrogen peroxide; MDA = malondialdehyde; SOD = superoxide dismutase; POD = peroxidase; CAT = catalase.
